# Immunization with a Recombinant, *Pseudomonas fluorescens*-Expressed, Mutant Form of *Bacillus anthracis*-Derived Protective Antigen Protects Rabbits from Anthrax Infection

**DOI:** 10.1371/journal.pone.0130952

**Published:** 2015-07-24

**Authors:** Matthew D. Reed, Julie A. Wilder, William M. Mega, Julie A. Hutt, Philip J. Kuehl, Michelle W. Valderas, Lawrence L. Chew, Bertrand C. Liang, Charles H. Squires

**Affiliations:** 1 Lovelace Biomedical and Environmental Research Institute (LBERI), Preclinical Drug Development, 2425 Ridgecrest Drive SE, Albuquerque, New Mexico, United States of America; 2 Pfenex Inc., 10790 Roselle St., San Diego, California, United States of America; Loyola University Chicago, UNITED STATES

## Abstract

Protective antigen (PA), one of the components of the anthrax toxin, is the major component of human anthrax vaccine (Biothrax). Human anthrax vaccines approved in the United States and Europe consist of an alum-adsorbed or precipitated (respectively) supernatant material derived from cultures of toxigenic, non-encapsulated strains of *Bacillus anthracis*. Approved vaccination schedules in humans with either of these vaccines requires several booster shots and occasionally causes adverse injection site reactions. Mutant derivatives of the protective antigen that will not form the anthrax toxins have been described. We have cloned and expressed both mutant (PA SNKE167-ΔFF-315-E308D) and native PA molecules recombinantly and purified them. In this study, both the mutant and native PA molecules, formulated with alum (Alhydrogel), elicited high titers of anthrax toxin neutralizing anti-PA antibodies in New Zealand White rabbits. Both mutant and native PA vaccine preparations protected rabbits from lethal, aerosolized, *B*. *anthracis* spore challenge subsequent to two immunizations at doses of less than 1 μg.

## Introduction

The gram-positive bacterium *Bacillus anthracis* is regarded as one of the most serious of all bioterror threats because of the persistence and ease of dispersion of *B*. *anthracis* spores as well as the rapid onset and lethality of disease resulting from spore inhalation [1]. After uptake of *B*. *anthracis* spores into the lungs, the spores are trafficked to lymph nodes where they germinate, enter the bloodstream and produce large quantities of anthrax toxins, which then play critical roles in disease progression, pathology, and lethality [2].

Anthrax toxins are composed of binary combinations of three proteins: protective antigen (PA), lethal factor (LF), and edema factor (EF) [3]. PA, the cell receptor-binding derivative of the toxin, combines with either LF to form lethal toxin (LT) or EF to form edema toxin (ET). Because of the central role that the toxins play in disease progression, most anthrax vaccines under development are based on neutralization of PA, the common, non-toxic component of LT and ET [4]. PA-based vaccines include Anthrax Vaccine Adsorbed (AVA or Biothrax), which is a cell-free filtrate of an avirulent, nonencapsulated variant of a *B*. *anthracis* culture that contains PA as the principal immunogen [5]. Other anthrax vaccines under development are composed of purified forms of recombinant PA (rPA) formulated with alum [6–9].

Recombinant PA manufacturing and alum-based formulations have been reported to be hampered by stability, potentially due to proteolytic sites on the rPA molecule [10]. A mutant form of PA (PA SNKE167-ΔFF-315-E308D, or mrPA) has been reported to have equivalent immunogenicity and increased stability vs. native (wtrPA, wild type) rPA [9,11]. Similar mutant isoforms have also shown wtrPA-equivalent preclinical immunological responses vs. wtrPA [12]. mrPA has two site mutations that remove proteolytically sensitive sites, altering residues RKKR at positions 164 to 167, to SNKE, and deleting residues FF at positions 314 to 315. Removal of the furin sensitive site RKKR prevents the PA from assuming its heptameric form that is responsible for pore formation and toxin action. Additionally, these mutations render the molecule more stable during post-expression purification steps [11]. The objective of this study was to test the feasibility to utilize this recombinant mrPA as an alternative to wtrPA in a subunit vaccine by comparing immunogenicity, toxin neutralization capacity, and efficacy of prototype alhydrogel-based vaccines of both wtrPA and mrPA proteins expressed and purified from the novel host system, *Pseudomonas fluorescens* [13]. The *P*. *fluorescens* system has proven to be a high yield expression system and to provide an excellent source (multiple grams of active protein expressed per liter in fermentation) of both the wtrPA and mrPA molecules for the studies reported herein (J. Allen, Pfenex Inc, Personal Communication). Other reports of immunogenicity of this mutant protein have come from studies in which the mrPA was prepared from derivatives of *B*. *anthracis* [9,11]. The series of studies reported here shows that mrPA prepared from this productive recombinant source induces a highly immunogenic and protective response in NZW rabbits, a species and strain commonly chosen to represent potential safety, immunogenicy, and efficacy of vaccines and rPA in humans.

## Materials and Methods

### Recombinant Production of Native and Mutant Protective Antigens

Genes encoding both the native and mutant forms (PA SNKE167-ΔFF-315-E308D) of PA were cloned into expression plasmids and transformed into derivative strains of *Pseudomonas fluorescens* strain MB101 [13]. Purified native (or wild type, wtrPA) and mutant PA (mrPA) were prepared by standard methods following fermentation of *P*. *fluorescens* expression strains including mircofluidic cell lysis, lysate clarification by centrifugation and filtration, followed sequentially by ion exchange and hydrophobic interaction chromatography and final filtration steps [J. Allen and D. Retallack (Pfenex Inc), personal communication].

### Vaccines and Formulation

wtrPA and mrPA products were formulated (Ajinomoto/Althea Technologies, San Diego, CA) to contain 1.0 mg/mL aluminum, added as Alhydrogel (InvivoGen, San Diego, CA) in Dulbecco’s phosphate buffered saline (DPBS). Dosage forms were prepared as follows: wtrPA formulations with 20 μg/mL, 5 μg/mL, and 1.25 μg/mL rPA protein in Alhydrogel; mrPA formulations with 20 μg/mL,5 μg/mL, and 1.25 μg/mL mrPA protein in Alhydrogel. The dosing solutions were prepared aseptically as 1 mL of total sample in glass vials and stored refrigerated (2–8°C). The positive control article, Anthrax Vaccine Adsorbed (AVA, trade name BioThrax), Lot FAV363, was characterized by its manufacturer (Emergent BioDefense Corporation, Lansing, MI). Negative control formulations were DPBS containing Alhydrogel. Lots of mrPA and wtrPA vaccines used for this study were characterized for activity by a LBERI Good Laboratory Practice (GLP 21CFR) validated potency assay (mouse anti-PA antibody ELISA and Toxin Neutralization Assay [TNA]) utilizing serum from immunized AJ mice [14,15].

### Rabbits

Forty-five male and 45 female New Zealand white (NZW) rabbits, certified *Pasteurella*-free (Covance Inc. Princeton, NJ), were placed on study. The rabbits weighed between 2.57–3.33 kg (females) and 2.45–3.29 kg (males) and were approximately 21–23 weeks old on Day 0 (study start). Rabbits were identified by subcutaneously placed Biometric Data Systems telemetry chips (Seaford, DE) and randomized into study groups using a validated computerized data acquisition system (Provantis, Instem LSS Ltd., Staffordshire, England) based on weight. Body weights of individual animals were ±20% of the group mean for each gender and groups were approximately equal mean body weights at study start prior to vaccination. Rabbits were housed individually in stainless steel/plastic cages in temperature and humidity controlled rooms on a 12h light cycle and fed Harlan Teklad Global Diet 2031C (Madison, WI) with free access to water. Prior to pathogen challenge, all test rabbits were conditioned and acclimated to restraint by being placed into rabbit exposure boxes for 10 ± 5, 30 ± 5 and 60 ± 5 minutes on separate days. The last conditioning session occurred within three days of exposure. All animimal procedures were carried out in strict accordance with the recommendations in the *Guide for the Care and Use of Laboratory Animals* of the National Institutes of Health. The protocol was approved by the Lovelace Respiratory Research Institute Animal Care and Use Committee. Animals were observed at least twice per day (morning and afternoon) prior to *B*. *anthracis* spore challenge and three times per day after challenge (see below) for signs of morbidity and mortality. Based on individual animal condition, additional observations for morbity and mortality occurred at the discretion of the Study Director in consultation with technical and veterinary staff. Examinations were oriented toward (1) identifying dead or moribund animals, and (2) documenting the onset of any abnormal clinical signs. The Study Director in consultations with the veterinary and technical staff made decisions regarding the euthanasia of moribund animals. Criteria for moribund status included severe respiratory distress, persistent recumbency and weakness, unresponsiveness to touch or external stimuli, extreme weight loss, or a combination of these observations. When morbidity was observed, all feasible actions were taken to limit pain and suffering by euthanizing animals by intravenous (IV) injection of an overdose of a barbiturate based sedative (Euthasol, Virbac, Ft. Worth, TX). Due to the nature of the study investigating the efficacy of a vaccine, no analgesics or anesthetics could be administered.

### Vaccinations and Study Design

The study design and endpoint analyses are described in Tables 1 and 2. Animal groups were derived from similar sample sizes utilized in previous reports of rPA and alum-based experimental animal vaccinations, immunogenicity, antibody neutralization, and efficacy/ protection endpoint determinations [14–17]. Groups of five male and five female New Zealand white (NZW) rabbits were vaccinated via intramuscular (IM) injection (hind leg) on Days 0 and 28 with 0.5 mL of one of six lots of wtrPA or mrPA as described above. Vaccinations were performed by group on each vaccination day. An AVA positive control group (human dose, 0.5 mL) and a vehicle control group were also included and dosed (0.5 mL, IM) on Days 0 and 28. An untreated control group was also included. Rabbits surviving to study conclusion were observed for 84 days following initial vaccination. All rabbits survived through the 28-day blood draw. Ten weeks after initial vaccination, rabbits were challenged with a target dose of 200 ± 50 LD_50_ of *B*. *anthracis* Ames spores (1.1 X 10^5^) [16,18]. Note: Two rabbits (one mrPA-10 and and one wtrPA-10) died or were euthanized just prior or just after *B*. *anthracis* challenge from non-infection related causes (n = 9 reported in some endpoints). Blood was collected from the marginal ear vein or central ear artery for all blood-associated endpoints. Serum was retained for the TNA and the ELISA at a time point pre-vaccination (baseline); at Days 14, 28, 42, and 65 following initial vaccination; and at euthanasia. Blood was collected on Days 65, 72, and 74 post-challenge and at euthanasia for hematology and clinical chemistry endpoints. Blood was also collected on Days 65, 71 through 74 and at post-challenge euthanasia for bacteriology. Sera were isolated on Days 65, 71 through 74 and at post-challenge euthanasia to detect PA by the electrochemiluminescence assay (ECL). Following challenge, rabbits that were found dead or euthanized were subject to a limited necropsy and samples were collected for bacteriology and histopathology assessments.

**Table 1:** Experimental Design—Vaccination^a^.

		Test Material				
Group	Test Material (Formulation Concentration, μg/ mL)	Target Dosage	Route	Vaccination Schedule	Dose Volume	Number of Rabbits^b^
1	Control	None	None	None	None	5 / 5 = 10
2	Vehicle Control	(0 μg)	IM	Day 0 and 28	0.5 mL	5 / 5 = 10
3	wtrPA- 20	High (10 μg)	IM	Days 0 and 28	0.5 mL	5 / 5 = 10
4	wtrPA- 5	Mid (2.5 μg)	IM	Days 0 and 28	0.5 mL	5 / 5 = 10
5	wtrPA- 1.25	Low (0.625μg)	IM	Days 0 and 28	0.5 mL	5 / 5 = 10
6	mrPA-20	High (10 μg)	IM	Day 0 and 28	0.5 mL	5 / 5 = 10
7	mrPA-5	Mid (2.5 μg)	IM	Day 0 and 28	0.5 mL	5 / 5 = 10
8	mrPA-1.25	Low (0.625μg)	IM	Days 0 and 28	0.5 mL	5 / 5 = 10
9	AVA	Human dose	IM	Days 0 and 28	0.5 mL	5 / 5 = 10
						**Total = 45 / 45 = 90**

^a^Animals vaccinated (0.5 mL per injection) with test or control article on Days 0 and 28.

^b^Equivalent numbers per sex, 5 males and 5 females per group.

**Table 2:** Experimental Procedures and Schedule.

	Study Day														
Procedure	Pre	0	7	14	21	28	35	42	Pre	70	71	72	73	74	84
Clinical Observations^a^	X	← X (2x daily) →									X (3x daily) X				
Body Weight^b^	X	← X (weekly) →													
Vaccination^c^		X				X									
Blood (ELISA, TNA)^d^	X			X		X		X	X						X
Challenge^e^										X					
Blood (Hematology)^f^									X			X		X	X
Blood (Clin.Chem.)^g^									X			X		X	X
Blood (Bacteriology)^h^									X		X	X	X	X	X
Blood (ECL)^i^									X		X	X	X	X	X
Target Blood Volume (mL)	5			5		5		5	8		2	4	2	4	9
Necropsy	X^j^										X^k^				

^a^Thrice daily observations performed on Days 72–74. Twice daily observations performed other days.

^b^Body weight obtained at randomization, on Day 0 and weekly thereafter.

^c^Animals vaccinated via intramuscular injection on Day 0 or Days 0 and 28 (see Table 1).

^d^Blood collected and sera isolated for TNA and ELISA on Days -6, 14, 28, 42 and 65 ± 4, when moribund euthanized or at terminal euthanasia (Day 84).

^e^Rabbits challenged with 200 x ± 50 LD_50_ *B*. *anthracis* (Ames) spores. The published inhalation LD_50_ for NZW rabbits is 1.1 x 10^5^ spores.

^f^Blood collected for CBC and differential.

^g^Blood collected and sera isolated for clinical chemistry parameters.

^h^Blood collected for quantitative bacteriology.

^i^Blood collected and sera isolated for electrochemiluminescence assay (ECL).

^j^Moribund euthanized or found dead rabbits received a limited gross necropsy.

^k^Euthanized or found dead rabbits received a gross necropsy and select tissues collected for bacteriology or histopathology.

### Enzyme-linked Immunosorbent Assay (ELISA)

Sera collected pre-vaccination and on Days 14, 28, 42, 65 and 84 were assayed for the presence of anti-PA antibodies by an Lovelace Respiratory Research Institute-developed Good Laboratory Practice (GLP 21 CFR) validated ELISA following general methods [16]. Sera collected at necropsy were 0.2 μM filter-sterilized prior to analyses. Antibodies to PA were measured in 96-well format plates (NUNC flat-bottomed wells; ThermoFisher, Waltham, MA) coated with 1 μg/mL of rPA diluted in Phosphate Buffered Saline (PBS) in a volume of 100 μL per well, sealed and incubated at 4°C for 12–18 hours. After incubation, plates were washed six times with wash buffer (0.1% Tween 20 in PBS) using a Bio-Tek ELx405 plate washer (Winooski, VT). Serum samples were two-fold serially diluted in assay buffer (5% non-fat dry milk, 0.1% Tween 20; PBS) following an initial 1:250 dilution in assay buffer. Single samples were added to the plate at a volume of 100 μl per well and incubated for one hour at 37°C. Following six washes, 100 μL of alkaline phosphatase-labeled goat anti-rabbit IgG (H+L-specific Catalog # 4751–1516; Kirkegard & Perry Laboratories, Gaithersburg, MD) diluted 1:2000 in assay buffer was added to each of the wells and incubated for one hour at 37°C. After six washes, 100 μl of 4-Nitrophenphenyl phosphate disodium salt hexahydrate in detection buffer (12.1% Tris (hydroxymethl) animomethane in PBS) was added to each of the wells and incubated covered for 30 minutes at 37°C. Plates were read at 405 nm using a Bio-Tek μQuant microplate reader and the data analyzed using Gen5 Data Analysis Software (Bio-Tek). Logistic regression analysis based on a 4-paramater curve was performed to determine the antibody concentration of various dilutions of test samples based on a standard curve constructed from pooled rabbit sera immunized with rPA with a concentration of 480 μg/mL anti-PA IgG [NR-3839; Biodefense and Emerging Infections Research Resources Repository (BEI), Manassas, VA]. At least five of seven data points in a two-fold dilution series were used to construct the standard curve based on the best R2 value. Only samples that fell between the upper and lower asymptotes of the 4-paramater curve were analyzed. If multiple dilutions of unknown samples interpolated to the standard curve, the μg/mL assigned was an average of those returned from each dilution. The lower limit of detection was determined by using the greatest dilution of rabbit reference serum that interpolated accurately (+/- 20%) to the standard curve and was at least three times the O.D. of the blank wells multiplied by the lowest dilution of the rabbit reference sera (1:250). Samples interpolating below the limit of detection were assigned that limit of detection multiplied by the lowest dilution of that sera.

### Toxin Neutralization Assay (TNA)

Sera collected pre-vaccination and on Days 14, 28, 42, 65 and 84 were assayed utilizing a Good Laboratory Practice (GLP 21 CFR) validated method for the presence of functional antibodies capable of neutralizing the toxic activity of *B*. *anthracis* lethal toxin based on that described [15]. Sera collected at necropsy were 0.2 μM filter-sterilized prior to analyses. J774A.1 mouse macrophage cells (American Type Culture Collection, Manassas, VA) were plated at 4 x 10^5^ cells per mL of maintenance medium (Dulbecco’s Modified Eagles medium with high glucose [4.5 g/L], and L-glutamine) in a 96-well flat bottom cell-culture plate (Costar, Corning, Tewksbury MA). The media was supplemented with 5% Fetal Bovine Serum, 1 mM sodium pyruvate, 100 units/mL penicillin and 100 μg/mL streptomycin sulfate, and 10 mM HEPES buffer, and the plates were incubated for 17–19 hours at 37°C with 5% CO_2_. Anthrax lethal toxin was made by the addition of rPA (BEI: NR-140; 0.1 μg/mL) and recombinant Lethal Factor (rLF; BEI: NR-4367; 0.08 μg/mL) to maintenance medium. Sera from the experimental rabbits was diluted 1:100 in maintenance medium and then two-fold serially diluted in a 96-well round bottom plate (Costar) to a final volume of 75 μL per well. An equal amount of lethal toxin was added to each well, except the reference row which received maintenance medium (75 μL) only. The positive control rabbit reference serum (BEI; NR-3839) was diluted 1:5 in maintenance medium prior to the 1:100 dilution for a working dilution of 1:500. The lethal toxin and rabbit sera were incubated together at 37°C for 30 minutes. The medium was removed from the cells and was replaced with 100 μL of dilutions of the toxin/neutralizing antibody mixtures, after which incubation proceeded as above for 4 hours. To assess cell viability, 25 μL of tetrazolium salt 3-[4,5-dimethylthiazol-2-yl]-2,5-diphenyltetrazolium bromide (MTT; 5 mg/mL) was added to each of the wells at the end of the 4 hour incubation and the plates were then incubated as above for an additional 2 hours. Afterwards, 100 μL of solubilization buffer (50% dimethyl formamide, Sigma with 200 mg/mL sodium dodecyl sulfate) was added to each of the wells and the final incubation proceeded as above for 16–20 hours. Plates were read at 570 nm using a Bio-Tek μQuant microplate reader and the data were analyzed using Gen5 Data Analysis Software (Bio-Tek). The ED_50_, the reciprocal of the dilution that inhibits 50% of the cell death due to the lethal toxin, was obtained by analysis of the 4-parameter curve. The Neutralization Factor (NF_50_) was determined as the ratio of the test sample ED_50_ to ED_50_ of the positive control reference serum.

### *B*. *anthracis* Spore Preparation

*B*. *anthracis* Ames spores used for rabbit inhalation challenge studies were prepared essentially as described [17]. Briefly, the *B*. *anthracis* Ames strain was propagated at 34 ± 2°C in a shaker incubator for 48 hrs in Modified Schaeffer’s Medium (2xSG). Spores were heat-shocked (65°C for 45 minutes), harvested by centrifugation (washed three times/ centrifuged-isolated in sterile water for injection), suspended in sterile water for injection and kept at 4 ± 2°C until use. Spores were heat-shocked a second time prior to use. Spore purity was verified on tryptic soy blood agar, MacConkey’s agar and phenylethyl alcohol agar by incubation at 37°C for up to 48 hrs. Spore lot titer was determined from serial dilutions of the spore preparation plated on tryptic soy agar (TSA) by incubation at 37°C for up to 48 hrs. Spore content was microscopically verified as >95% and presence of virulence plasmids PX01 and PX02 were confirmed by PCR.

### Inhalation Challenge

On Day 70 post-vaccination rabbits received a targeted dose of 200 ± 50 LD_50_ of *B*. *anthracis* Ames by nose-only exposure [16,18]. The spores were nebulized in a Collison nebulizer (MRE-3 jet, BGI, Inc., Waltham, MA). An all glass impinger (AGI) sample of the bioaerosol was obtained to characterize presented inhalation dose. The un-anesthetized rabbit rested in a purpose built plethysmography box juxtaposed for nose only exposure essentially as described [16]. The rabbit’s breathing frequency, tidal volume, and minute volume were each measured during the exposure [16,17,19]. The duration of the exposure was based on the total volume of air inhaled by the rabbit. Bacterial aerosol concentrations were confirmed by quantitative bacterial culture of AGI samples using standard dilution plating on TSA plates. Cultures were incubated at 37 ± 2°C for 18–24 hours prior to counting. The target particle size of the aerosol was 1 to 3 μm and was determined using a GRIMM Portable Aerosol Spectrometer Model 1.109 [GRIMM Aerosol Technik GmbH & Co. KG, Ainring, Germany] for 0.5 to 20 μm particles. Aerosol dose was calculated after direct measurement of inhaled volume and aerosol concentration using the following formula: Dose = (C × V), where C is the concentration of viable pathogen in the exposure atmosphere, and V is the volume inhaled.

### Hematology and Serum Chemistries

Blood was collected on Days 65, 72, 74 and 84 post vaccination for complete blood count (CBC) and sera isolated for a standard panel of serum parameters. CBC determinations were made using an Advia 120 (Siemens AG, Erlangen, Germany). Serum chemistries were determined using a Hitachi 911 Chemistry Analyzer or a Hitachi Modular Analytics Clinical Chemistry System (Roche Diagnostics, Indianapolis, IN).

### Electrochemiluminescence Assay (ECL)

Sera collected on Days 65, 71 through 74 and at euthanasia was assayed for the presence of anthrax protective antigen in serum using an in-house, GLP validated, qualitative ECL assay [20]. This assay measured the PA in serum using Meso-Scale Discovery (MSD, Rockville, MD) ECL technology and the MSD *B*. *anthracis* PA assay kit. The PA was detected by the addition of the MSD detector antibody (STAG [sulfonated derivative of Ruthenium (II) tris-bipyridine tag]-labeled anti-PA antibody). The amount of PA present in the sample was determined by the amount of light that is emitted upon electrochemical stimulation of the STAG initiated at the electrode surface of the kit’s microplate.

### Bacteriology

Blood was collected on Days 65, 71 through 74 and 84 for determination of qualitative or quantitative (Day 84) bacteriology. No baseline serological analyses were reported. All rabbits that were found dead or euthanized post-challenge were necropsied and had select tissues assayed for quantitative bacteriology. For qualitative assessments, collected blood was plated onto a single, sterile 90 mm tryptic soy agar (TSA) plate and incubated at 37°C for 16–24 hr, after which the presence or absence of *B*. *anthracis* colonies was determined. For quantitative assessments, blood and tissue samples were serially diluted in sterile 1% peptone. From each dilution, 100 μL was removed and plated onto sterile 90 mm TSA plates in triplicate and allowed to incubate at 37°C for 16–24 hr. *B*. *anthracis* titer (colony forming units [CFU]/mL) was calculated:

$$\frac{CFU}{mL}\;=\;mean\;CFU\;count\times Total\;dilution\;factor$$

The total dilution factor included the media plate inoculum volume of 100 μL (i.e., an additional 10-fold dilution was included in the final calculations to achieve the titer in terms of CFU/mL).

### Necropsy and Pathology

Rabbits surviving to the scheduled sacrifice or determined to be moribund were euthanized by intravenous (IV) injection of an overdose of a barbiturate based sedative (Euthasol, Virbac, Ft. Worth, TX). Lung, spleen, heart, and tracheal-bronchial lymph nodes (when present) were collected for bacteriology and histopathology. Tissue sections were fixed in 10% neutral-buffered formalin (NBF), cut, mounted on slides, stained with hematoxylin and eosin (H&E) and microscopic findings evaluated for incidence and severity by a board certified veterinary pathologist. Findings were given a score from 1 to 4, based upon a subjective assessment of the overall severity in the tissue present on the slide (1 = minimal; 2 = mild; 3 = moderate; 4 = marked). Where appropriate, an assessment of distribution was also made (F = focal, M = multifocal, D = diffuse, Ws = widespread, Lx = locally extensive). A qualitative assessment of bacterial burden was also made where appropriate.

### Statistics and Primary Experimental Outcomes Assessed

Survival, immunogenicity, antibody nuetralizations were the primary outcomes assessed for this study. The number of rabbits dying and mean time to death and/or mean survival time was determined for each group. Survival analysis was tested by log-rank (Mantel-Cox) test analysis [Prism 5.04 (GraphPad Software, Inc.)]. In addition, survival at study end for each experimental group was analyzed by Chi-square test. The body weight/change data were statistically analyzed for each time point for differences in treatment group by analysis of variance (ANOVA) and, if appropriate, Dunnett's test. A p value of ≤ 0.05 was considered significant. TNA and ELISA data were log-transformed prior to being statistically analyzed by repeated-measures ANOVA to determine whether any differences exist between the experimental groups. A p < 0.05 was considered to be significant. If significant, a post hoc multiple comparison t-test with Bonferroni’s adjustment was performed. A significant p value was dependent on whether it is smaller than 0.05/ (number of tests). Statistical analyses were conducted in Statview 5.0.1 (SAS, Cary NC). Data is presented as a group with a geometric mean of 95% confidence intervals.

## Results

### Induction and Measurement of Anti-PA Antibodies

**Fig 1:**
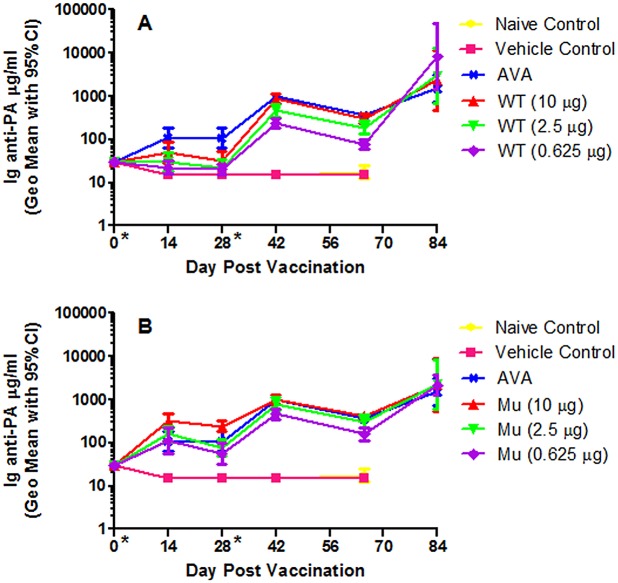
Ig anti-PA ELISA values (μg/mL). (A) generated from vaccinated wild type and (B) mutant rPA (geometric mean with error bars representing the 95% confidence interval).

A rabbit immunogenicity and *B*. *anthracis* spore challenge study was designed (Tables 1 and 2) to test the effectiveness of both the wtrPA and mrPA molecules at a broad range of doses (dosed at over a 16-fold range) in a two dose vaccine administration regimen to induce an anti-PA antibody response. The approved anthrax vaccine (Biothrax) administered at the human dose, 0.5 mL, served as the positive control. Results for the anti-PA ELISA analyses performed on serum samples collected as outlined in Table 2 are shown in Fig 1 and Tables 3 and 4. Multiple vaccine groups’ immunogenicity responses over time (curve response) were significantly different from other groups when compared by repeated-measures ANOVA and Bonferroni/Dunn post-hoc test when IgG anti-PA values from Days -1 through 65 were analyzed (Table 4). The highest dose of wtrPA (10 μg) induced a significantly greater immune response than did the two lower doses (2.5 and 0.625 μg) when considering the blood draws Days -1 through 65, Table 4. The highest dose of mrPA induced a significantly higher total antibody titer than either of the two lower doses. The high dose of the mrPA also induced a significantly higher anti-PA response than the human dose of AVA. The 2.5μg mrPA dose was not statistically different from AVA. The mrPA outperformed the wtrPA at all comparable doses.

**Table 3:** Study Summary of Fate and Immune Status Relevant to B. anthracis Challenge on Day 70.

		IgG anti- PA (μg/mL) Geometric Mean ± C.I.		TNA ED50 Geometric Mean ± C.I.	
Test Group (μg rPA)	Survival (Alive/Total)	Day 42	Day 65	Day 42	Day 65
Naïve Control	0% (0/10)	15 (15, 15)^a^	17 (12, 24)	1 (1, 1)^b^	1 (1, 1)
Vehicle Control (0 μg rPA)	0% (0/10)	15 (15, 15)	15 (15, 15)	1 (1, 1)	1 (1, 1)
wtrPA 10	100% (9/9)	869(674, 1121)	303(240, 381)	27324(18802, 39709)	6608(4522, 9656)
wtrPA 2.5	90% (9/10)	456(325, 640)	190(136, 264)	17670(11320, 27583)	4282(2310, 7937)
wtrPA0.625	80% (8/10)	234(182, 301)	78(61, 99)	8992(6394, 12644)	1176(499, 2773)
mrPA 10	100% (9/9)	1008(800, 1271)	416(338, 512)	37725(20959, 67903)	13,437(7561, 23882)
mrPA 2.5	100% (10/10)	800(553, 1158)	306(217, 432)	22176(14214, 34598)	8695(4706, 16065)
mrPA 0.625	100% (10/10)	456(335, 621)	159(114, 224)	13674(9504, 19676)	2947(1366, 6358)
AVA	100% (10/10)	974(802, 1183)	363(298, 443)	17523(10933, 28085)	4798(2975, 7737)

^a^Lower limit of quantitation < 30 μg/mL.

^b^Samples which returned an incalculable ED50 (by Gen 5 software) were assigned an ED50 value of 1.

**Table 4:** p Values Associated with Comparison of Ig anti-PA Values between Vaccine Groups as Analyzed by Repeated Measures ANOVA.

Vaccine Group	WTrPA10	WTrPA2.5	WTrPA0.625	mrPA10	mrPA2.5	mrPA 0.625	AVA
wtrPA 10 μg	–						
wtrPA 2.5 μg	< .0011↓	–					
wtrPA 0.625 μg	< .0001↓	NS	–				
mrPA 10 μg	< .0001↑	< .0001↑	< .0001↑	–			
mrPA 2.5 μg	NS	< .0001↑	< .0001↑	< .0001↓	–		
mrPA 0.625 μg	NS	< .0008↑	< .0001↑	< .0001↓	NS	–	
AVA	< .0002↑	< .0001↑	< .0001↑	< .0009↓	NS	< .0003↑	–

Ig anti-PA levels from blood draws between day -1 and 65 were analyzed by repeated measures ANOVA.

The Ig anti-PA response of non-vaccinated groups (None and Vehicle, not shown above) were significantly lower (p < 0.0001) than every other group except each other.

^↓^Indicates the Ig anti-PA response of the vaccine group listed on the left is significantly less than the corresponding vaccine group listed on the top of the table.

^↑^Indicates the Ig anti-PA response of the vaccine group listed on the left is significantly greater than the corresponding vaccine group listed on the top of the table.

NS, no statistical significance between the two groups.

Subsequent to the boost vaccination delivered on Day 28, anti-PAantibodies increased in all dose groups to a similar level prior to challenge. The levels of total anti-PA antibody increased post-aerosol challenge in all vaccine groups, Fig 1. Often the highest post-challenge responses were measured in those rabbits vaccinated with the lowest dose of rPA.

### Induction and Measurement of Toxin Neutralizing Antibodies

**Fig 2:**
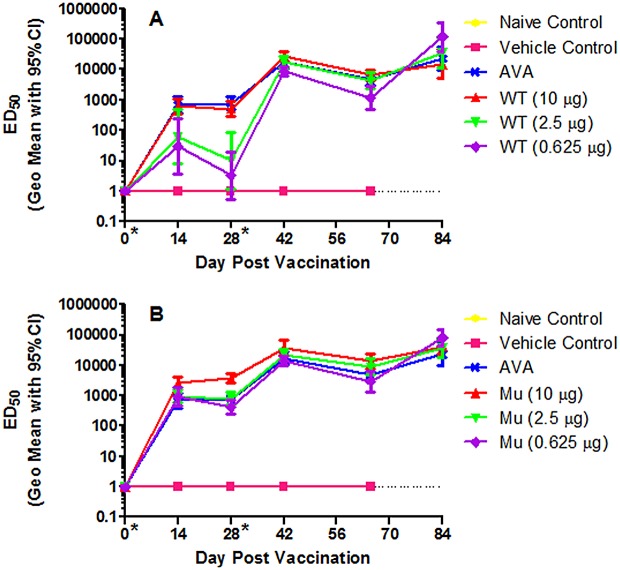
rPA antibody-specific Toxin Neutralization Assay (TNA) and ED50 values (μg/mL). (A) generated from vaccinated wild type and (B) mutant rPA (geometric mean with error bars representing the 95% confidence interval).

In addition to the characterization of the appearance of total anti-PA antibodies (described above), anthrax holotoxin neutralizing anti-PA-antibodies were also measured by a toxin neutralization assay (Tables 1 and 2). The approved anthrax vaccine (Biothrax) administered at the human dose, 0.5 mL, once again served as the positive control. Fig 2 and Tables 3 and 5 illustrate the results of the TNA for each vaccine group. Multiple vaccine groups were significantly different from other groups when time-based ED_50_ data were analyzed by repeated-measures ANOVA and Bonferroni/Dunn post-hoc test over Days -1 through 65, Table 5. The highest dose of wtrPA induced a significantly greater immune response than did the two lower doses of wtrPA when considering the blood draws Days -1 through 65. The highest dose of mrPA was similar to the 2.5μg dose of mrPA, but induced a significantly higher amount of functional antibody than either the lowest dose of mrPA or both the 2.5 μg and 0.625 μg doses of wtrPA. Also, higher ED_50_ values were measured in rabbits from the highest mrPA dose group (10 μg, p = 0.0002) as compared to AVA. The mrPA outperformed wtrPA at all comparable doses and the two lower doses of mrPA were equivalent and not statistically different from the highest wtrPA doses or AVA.

**Table 5:** p Values Associated with Comparison of ED50 Values between Vaccine Groups as Analyzed by Repeated Measures ANOVA

Vaccine Group	WTrPA10	WTrPA2.5	WTrPA0.625	mrPA10	mrPA2.5	mrPA0.625	AVA
wtrPA 10 μg	–						
wtrPA 2.5 μg	< .0001↓	–					
wtrPA 0.625 μg	< .0001↓	NS	–				
mrPA 10 μg	.0003↑	< .0001↑	< .0001↑	–			
mrPA 2.5 μg	NS	< .0001↑	< .0001↑	NS	–		
mrPA 0.625 μg	NS	< .0001↑	< .0001↑	< .0001↓	NS	–	
AVA	NS	< .0001↑	< .0001↑	.0002↓	NS	NS	–

TNA ED_50_ values from blood draws between day -1 and 65 were analyzed by repeated measures ANOVA.

The ED_50_ response of non-vaccinated groups (None and Vehicle, not shown) were significantly lower (p < 0.0001) than every other group except each other.

^↓^Indicates the ED_50_ response of the vaccine group listed on the left is less than the corresponding vaccine group listed on the top of the table.

^↑^Indicates the ED_50_ response of the vaccine group listed on the left is greater than the corresponding vaccine group listed on the top of the table.

NS, no statistical significance between the reactions of the two groups.

In general, an rPA dose response was observed prior to the Day 28 boost, particularly in the wtrPA dosed rabbits. After the Day 28 boost, the differences between doses of rPA, either wild type or mutant, became less apparent and the levels declined between Day 48 and 65 prior to challenge. Neutralizing antibody levels increased post-aerosol challenge in all vaccine groups as seen in Fig 2.

### Mortality Following B. anthracis Spore Challenge

**Fig 3:**
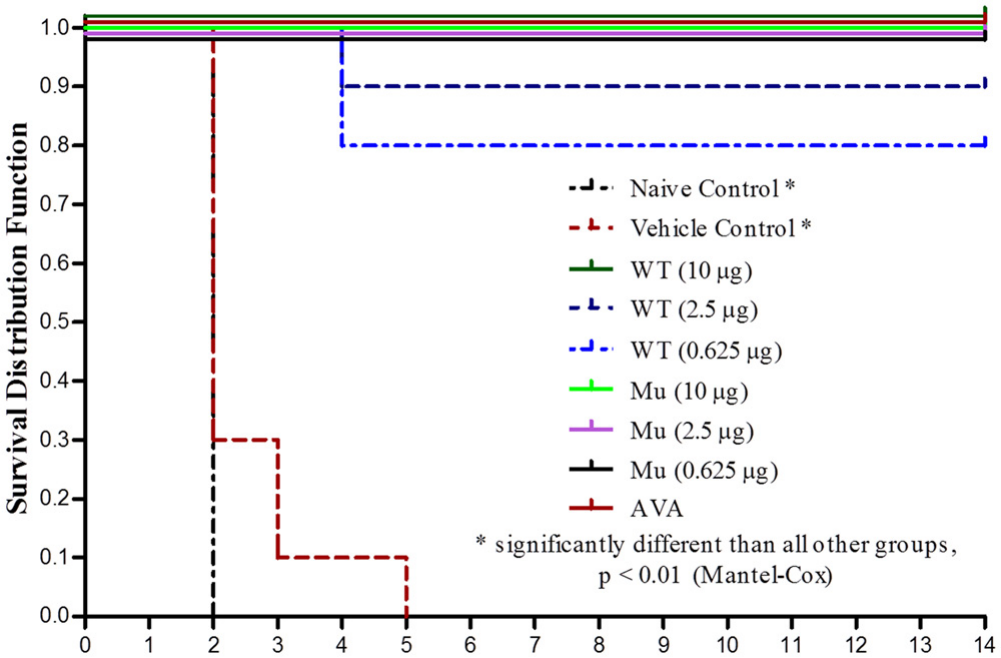
Kaplan-Meier Survival Curve of vaccinated, unvaccinated control, and sham vaccinated control rabbits.

Mortality data and Kaplan-Meier data are shown in Table 3 and Fig 3. All rabbits survived until challenge with the exception of single 10ug mrPA female rabbit that died during conditioning to inhalation exposure restraint. One 10 μg wtrPA rabbit was euthanized subsequent to challenge due to partial paralysis unrelated to exposure. All Naïve and Vehicle Control group rabbits died due to anthrax infection two to five days post-challenge (Days 72–75). One 1.25 and two 0.625 μg wtrPA-immunized rabbits died due to anthrax infection on Day 74. All 10 μg wtrPA, all mrPA vaccinated, and AVA rabbits survived challenge. There were statistically significant differences (p < 0.0001, Mantel-Cox; Chi-square) in survival and time to death for the Naïve and Vehicle Control groups compared to all other test groups. A statistically significant difference was not observed for the vaccinated groups [2.5 μg wtrPA and 0.625 μg wtrPA)] that had post-challenge deaths compared to the remaining test groups.

### Bacteriological Evaluation of Vaccinated Rabbits Challenged with *B*. *anthracis* Spores

Six of ten naïve control rabbits and eight of ten vehicle control rabbits were positive for bacteremia in the blood (data not shown). Of vaccinated rabbits, there were only three rabbits that demonstrated bacteremia at the initial blood (Day 71) draw following challenge, but this resolved in these rabbits by the next blood collection time point (Day 74) and the rabbits survived to Day 84. Of the three wtrPA-vaccinated rabbits that succumbed to disease, none had bacteremia present in any blood sample.

**Fig 4:**
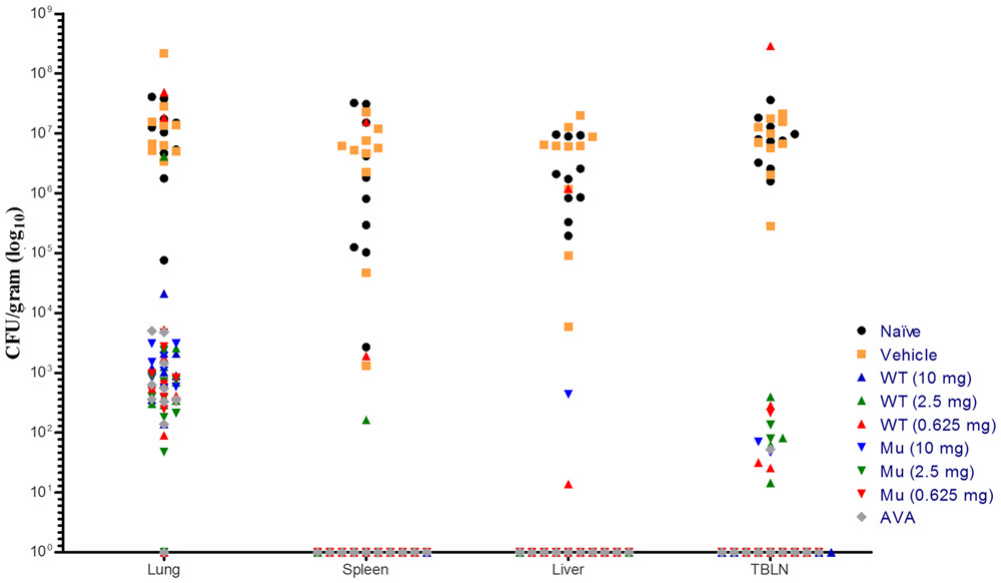
Recovery of *B*. *anthracis* from Select Tissues of vaccinated, unvaccinated control, and sham vaccinated control rabbits.

Negative control rabbits (unvaccinated and those vaccinated with vehicle) were shown to have high levels of tissue burden for all tissues tested, Table 6 and Fig 4. All other rabbits that succumbed to disease presented with marked tissue burden in the majority of tissues tested. In the rabbits that succumbed to disease, lung burden ranged from 7.56 x 10^4^ to 2.21 x 10^8^ CFU/mL. The rabbits who survived to study termination were found to have tissue burden below the detectable limits or, in a few cases, low tissue burden was seen in the lungs. In the three wtrPA vaccinated rabbits that succumbed to disease prior to study termination, high tissue burden was seen in the lung and low levels in the tracheobronchial lymph nodes (TBLN) and spleen. One of the animals that succumbed had high tissue burden in the lung as well as all other tissues collected. The rabbits in the mrPA vaccine groups all survived to study termination and any *B*. *anthracis* present in the tissues was at lower levels (less than 30 CFU/100 μL inocula) than wtrPA vaccine group rabbits.

**Table 6:** Summary of Recovery of *B*. *anthracis* from Select Tissues of Inhalational Spore Challenged New Zealand White Rabbits.

Dose^a^		Lung	Liver	Spleen	TBLN
**Naïve Control**	Mean^b^	7.13	6.25	6.00	6.87
	SD	—	0.600	1.293	0.408
	GeoM^c^	1.34 x 10^7^	1.79 x 10^6^	9.94 x 10^5^	7.38 x 10^6^
	N	1	10	10	10
**Vaccinated Control**	Mean	8.08	6.33	6.24	6.81
	SD	—	1.118	1.314	0.563
	GeoM	1.20 x 10^8^	2.14 x 10^6^	1.75 x 10^6^	6.41 x 10^6^
	N	1	10	10	10
**wtrPA 10 mg**	Mean	3.29	BDL	BDL^d^	BDL
	SD	—	BDL	BDL	BDL
	GeoM	1.95 x 10^3^	BDL	BDL	BDL
	N	1	10	10	10
**wtrPA 2.5 mg (2.5 mg)**	Mean	2.51	BDL	0.22	0.75
	SD	1.585	BDL	0.700	1.022
	GeoM	3.24 x 10^2^	BDL	6.65 x 10^−1^	4.57 x 10^0^
	N	7	10	10	10
**wtrPA 0.625 mg**	Mean	5.56	0.72	1.05	1.38
	SD	3.916	1.917	2.393	2.639
	GeoM	3.59 x 10^5^	4.28 x 10^0^	1.02 x 10^1^	2.31 x 10^1^
	N	2	10	10	10
**mrPA 10 mg**	Mean	2.03	0.26	BDL	0.35
	SD	1.755	0.835	BDL	0.744
	GeoM	1.05 x 10^2^	8.37E-01	BDL	1.25 x 10^0^
	N	3	10	10	10
**mrPA 2.5 mg**	Mean	3.40	BDL	BDL	0.40
	SD	—	BDL	BDL	0.853
	GeoM	2.49 x 10^3^	BDL	BDL	1.53 x 10^0^
	N	1	10	10	10
**mrPA 0.625**	Mean	2.67	BDL	BDL	0.23
	SD	0.005	BDL	BDL	0.736
	GeoM	4.71 x 10^2^	BDL	BDL	7.09 x 10^−1^
	N	2	10	10	10
**AVA (human dose)**	Mean	3.01	BDL	BDL	0.17
	SD	0.761	BDL	BDL	0.543
	GeoM	1.03 x 10^3^	BDL	BDL	4.85E-01
	N	3	10	10	10

^a^Treatment = Dose delivered intramuscularly (IM) on Days 0 and 28 in 0.5 mL volume

^b^Mean = Log_10_ (CFU/g + 1)

^c^Geometric mean of CFU/g

^d^BDL = Below detection limit (< 68–70 CFU/g)

### Clinical Pathology

Most elevated clinical pathology parameters were associated with rabbits that succumbed to challenge in the vehicle control and naïve animal groups (data not shown and no statistical analyses performed). Elevated parameters included blood urea nitrogen, creatinine, alanine aminotransferase, aspartate aminotransferase, alkaline phosphatase, lactate dehydrogenase, triglycerides and gamma glutamyltransferase.

Hematology parameters were generally unremarkable prior to challenge (data not shown and no statistical analyses performed). Subsequent to challenge white blood cell (WBC) counts increased in virtually all rabbits. In those rabbits that survived, WBC counts returned to normal by sacrifice.

### Pathology

The microscopic findings in the untreated and vehicle control rabbits were characteristic of anthrax and were pooled for baseline data, Table 7 [18]. Lungs retained bacteria in the septal capillaries and larger arteries and veins. Most rabbits had some degree of heterophilic inflammation and necrosis/fibrosis within the septal capillaries, along with more variable hemorrhage and edema. Of the tracheobronchial lymph nodes collected (11 of 20) and presumably not destroyed by anthrax infection, the majority exhibited necrosis, depletion of lymphocytes, and general inflammation. Bacteria were present in the lymph node sinuses and lymphatics as well as the blood vessels. Spleens were characterized by necrosis, depletion of the white pulp lymphocytes, and fibrosis and inflammation with necrosis of the red pulp. Bacteria were identified in the red pulp of all unvaccinated rabbits. Liver inflammation, fibrosis and necrosis along with associated cell loss occurred to some extent in unvaccinated-challenged rabbits. Bacteria were present in the blood vessels of the brain meninges in these animals.

**Table 7:** Incidence of Pulmonary Microscopic Findings in the Lungs of *B*. *anthracis* Spore Exposed Rabbits by Group (values represent the number of animals within a study group that possessed the finding).

	Group (μg)								
Number of Animals on Study:	Naïve Control	Vehicle Control	*wtrPA 10	wtrPA 2.5	wtrPA 0.615	mrPA 10	mrPA 2.5	mrPA 0.625	AVA
	10	10	10	10	10	9	10	10	10
**LUNG(S)**									
# Examined	10	10	10	10	10	9	10	10	10
# Within Normal Limits	0	0	0	0	0	1	0	0	0
**Congestion**	9	10	9	10	10	8	10	10	9
Minimal 1	0	0	3	3	2	1	1	0	1
Mild 2	1	1	3	5	6	4	6	5	4
Moderate 3	8	9	3	1	1	3	3	4	2
Severe 4	0	0	0	0	0	0	0	1	1
**Hemorrhage**	0	4	0	1	3	1	0	0	1
Minimal 1	0	1	0	0	0	1	0	0	0
Mild 2	0	3	0	0	1	0	0	0	1
Moderate 3	0	0	0	1	2	0	0	0	0
**Edema**	9	6	0	1	4	0	0	0	0
Minimal 1	2	1	0	0	1	0	0	0	0
Mild 2	4	3	0	0	1	0	0	0	0
Moderate 3	3	2	0	1	2	0	0	0	0
**Bacteremia**	10	10	0	0	1	0	0	0	0
Mild 2	0	3	0	0	0	0	0	0	0
Moderate 3	4	4	0	0	0	0	0	0	0
Severe 4	6	3	0	0	1	0	0	0	0
**nfiltration, Heterophils, Fibrin,**									
**±Necrotic Cell Debri; Interstitium; Capillary**	10	9	0	0	1	0	0	0	0
Minimal 1	6	1	0	0	0	0	0	0	0
Mild 2	3	8	0	0	1	0	0	0	0
Moderate 3	1	0	0	0	0	0	0	0	0
**Increased; Mucosa Associated Lymphoid Tissue**	0	0	9	10	8	8	10	10	10
Minimal 1	0	0	3	2	2	5	3	4	2
Mild 2	0	0	5	8	6	3	7	5	7
Moderate 3	0	0	1	0	0	0	0	1	1
**Infiltration, Lymphocytic,**									
**Histiocytic; Interstitium**	0	1	7	9	10	8	9	8	9
Minimal 1	0	0	6	3	3	4	5	5	4
Mild 2	0	1	1	5	5	4	4	2	5
Moderate 3	0	0	0	1	2	0	0	1	0
**Accumulation; Alveolus; Macrophage**	0	0	5	8	6	6	6	7	5
Minimal 1	0	0	4	4	1	6	5	5	4
Mild 2	0	0	0	3	4	0	1	1	1
Moderate 3	0	0	1	1	1	0	0	1	0
**Inflammation, Heterophils;**									
**Peribronchiolar; Lymphatic**	0	4	2	5	6	2	2	4	2
Minimal 1	0	0	1	0	0	1	2	3	1
Mild 2	0	3	1	3	3	1	0	1	1
Moderate 3	0	1	0	2	3	0	0	0	0
**Inflammation, Granulomatous;**									
**Lymphatic; Interstitium**	0	0	2	6	4	1	1	1	3
Minimal 1	0	0	2	4	0	1	1	0	1
Mild 2	0	0	0	2	3	0	0	0	2
Moderate 3	0	0	0	0	0	0	0	1	0
Severe 4	0	0	0	0	1	0	0	0	0
**Abscess**	0	0	0	1	2	0	0	1	0
Mild 2	0	0	0	1	1	0	0	1	0
Moderate 3	0	0	0	0	1	0	0	0	0
**Inflammation, Lymphocytic, Histiocytic; Pleura**	0	0	0	0	1	0	0	0	0
Mild 2	0	0	0	0	1	0	0	0	0
**Inflammation, Histiocytic, Heterophilic; Alveolus**	0	0	0	1	1	0	0	0	0
Moderate 3	0	0	0	0	1	0	0	0	0
Severe 4	0	0	0	1	0	0	0	0	0
**Bacteria; Lymphatic; Alveolus**	0	0	0	1	1	0	0	0	0
Moderate 3	0	0	0	0	1	0	0	0	0
Severe 4	0	0	0	1	0	0	0	0	0

*Rabbit included that was euthanized post exposure (9h) due to causes unrelated to treatment. All pathology findings were within normal ranges.

Microscopic findings in AVA-vaccinated rabbits were consistent with a response to a high dose of inhaled spores in immunized rabbits. Inflammatory cells were scattered throughout the pulmonary interstitium in most rabbits, and there was an increase in the number of alveolar macrophages, many of which contained cytoplasmic particles approximately 1–2 microns in diameter (possible spores). A few rabbits had foci of granulomatous inflammation associated with lymphatic vessels and/or heterophilic (rabbit neutrophil equivalent) inflammation associated with the conducting airways and lymphatics. All rabbits had an increase in the mucosal-associated lymphoid tissues of the airways and follicles with germinal centers in the tracheobronchial lymph nodes, and 8/10 had an increase in the paracortical zone lymphocytes and/or medullary plasma cells. Spleens and livers were generally unremarkable.

The microscopic findings in rabbits from all 10 μg wtrPA, all 2.5 and 10 μg mrPA, eight of ten 2.5 μg wtrPA, six of ten 0.625 μg wtrPA, and nine of ten 0.625 μg mrPA treated rabbits were generally equivalent to the AVA group and survived to Day 84 necropsy. In addition to an AVA-like inflammatory response, a single 0.625 μg mrPA, one 2.5 μg wtrPA and two 0.625 μg wtrPA treated rabbits additionally retained small inflammatory abscesses in a single lung lobe at Day 84. Of the 2.5 μg wtrPA and two 0.625 μg wtrPA treated rabbits that succumbed to *B*. *anthracis* spore exposure, the 2.5 μg wtrPA treated rabbit and one of the 0.625 μg wtrPA treated rabbits retained bacteria in the pulmonary lymphatics and alveolar spaces and generalized heterophilic inflammation. The remaining 0.625 μg wtrPA treated rabbit death had findings similar to unvaccinated rabbits.

### Electrochemiluminescence Assay (ECL) for Residual Circulating Protective Antigen

All animals lacked detectable circulating PA in the serum prior to challenge and almost 19/20 control rabbits were PA positive by Study Day 72. Untreated and vehicle-treated *B*. *anthracis* spore-exposed control animals remained PA positive until death. Subjects in vaccinated groups had sporadic, transient PA positive test samples; the 10μg wtrPA (3/10) and 2.5 μg wtrPA (4/10) had the highest number of subjects with a PA positive test sample, but all PA positive test subjects in vaccinated animals returned to a PA negative state by the end of the study.

## Discussion

In order to test the feasibility of developing a recombinantly-derived mutant form of *B*. *anthracis* protective antigen as a vaccine, the current study was undertaken to compare the efficacy of native, wtrPA and mutant, mrPA vaccines to protect rabbits from an aerosol challenge with *B*. *anthracis* spores. Animal numbers were statistically sufficient to answer this question and were consistent with previous literature-based investigations of rPA vaccines [21].

The mutant form of the rPA studied here may present production and functional advantages as a vaccine antigen. The mutant has had two proteolytically sensitive sites removed. These mutations may serve to protect the intact antigen product to proteolytic loss over long periods of storage, making it a better candidate for a stock-piled vaccine. Ramirez *et al*. (2002) have also found an increased yield of this protein compared to the native molecule when prepared from a recombinant *B*. *anthracis* host [11], presumably due to the mutations. Also, because the wtrPA must be cleaved to assume its active configuration, a proteolytically resistant form may have advantages for a post exposure prophylactic use. In such cases, the vaccine will not introduce any further component of the anthrax toxin to exposed individuals [22]. A 24-month stability study utilizing a GLP validated mouse potency assay is currently underway assessing mrPA and wtrPA proteins expressed and isolated from *P*. *fluorescens* and formulated in alum (data not shown). The data that will be derived from this study will reveal any differentiation of the stability of wtrPA vs. mrPA formulated with Alhydrogel.

Immunogenicity results from the initial preclinical work performed [11] as well as a Phase I clinical study [9] investigating the mrPA vs. wtrPA mirror those of the current study. The mrPA performed at least as well as the wtrPA and was comparable to AVA. ELISA and neutralizing antibody responses are very similar to those produced by the native protein.

In the current study, ELISA and TNA results from both rPA molecules were consistent with previously reported alum-based rPA anthrax vaccinations and challenge studies [21]. However, this study is the first to show a detailed comparative assessment of the immunogenicity, TNA response, and survival of rabbits vaccinated with alum formulations of mrPA vs. wtrPA derived from a novel recombinant source, *P*. *fluorescens*, and challenged with *B*. *anthracis* spores. mrPA generated circulating and neutralizing antibodies that were superior to all comparable doses of wtrPA and superior to the human dose of AVA when administered at the 10 μg/dose level. The mrPA and wtrPA comparative antibody response was reflected in trends observed in survival, general bacteremia and pathology, suggesting that the mrPA conferred superior protection compared to the wtrPA protein. Such mutants that cannot heptamerize have been shown to be more effective antigens. Perhaps this is because they disrupt normal cellular trafficking, making them more susceptible targets for the host’s immune system [23]. Bacteriology, pathology and survival also suggest that the antibodies produced by mrPA are at least as protective as the human dose of AVA. It is interesting to consider how these results could potentially be compared to long term rabbit vaccination and challenge studies, as well as human anti-PA IgG and TNA responses generated from AVA and other rPA-based vaccines under development. However, the 14-day post challenge sacrifice date used in the current study design was not long enough in duration to determine whether or not full *B*. *anthracis* lung clearance could be achieved at an associated TNA or anti-PA value.

In summary, these data demonstrate that recombinant mrPA and wtrPA produced in a novel expression system can be formulated with alum to achieve protection that is equivalent to the clinical administration of AVA. Under the conditions of this study, mrPA outperformed the wtrPA formulation and was equivalent to AVA at dose levels as low as 0.625 μg in a two dose regimen. These data combined with ongoing stability studies currently suggest that both the alum-formulated wtrPA and mrPA proteins expressed in *P*. *fluorescens* are viable vaccine candidates suitable for further development [24].
